# Investigation of Mechanical Properties of Polymer-Infiltrated Tetrapodal Zinc Oxide in Different Variants

**DOI:** 10.3390/ma17092112

**Published:** 2024-04-29

**Authors:** Franziska Scherer, Sebastian Wille, Lena Saure, Fabian Schütt, Benjamin Wellhäußer, Rainer Adelung, Matthias Kern

**Affiliations:** 1 Department of Prosthodontics, Propaedeutics and Dental Materials, School of Dentistry, Christian-Albrechts University at Kiel, Arnold-Heller-Straße 16, 24105 Kiel, Germany; swille@proth.uni-kiel.de (S.W.); benjamin.wellhaeusser@t-online.de (B.W.); mkern@proth.uni-kiel.de (M.K.); 2 Functional Nanomaterials, Institute for Materials Science, Kiel University, Kaiserstr. 2, 24143 Kiel, Germany; lms@tf.uni-kiel.de (L.S.); fas@tf.uni-kiel.de (F.S.); ra@tf.uni-kiel.de (R.A.)

**Keywords:** tetrapodal zinc oxide, PICN, BisGMA, TEGDMA, primer, flexural strength

## Abstract

The aim of this study was to evaluate the influence of weight ratio, the shape of the precursor particles, and the application of a phosphate-monomer-containing primer on the mechanical properties of polymer infiltrated ceramic networks (PICNs) using zinc oxide. Two different types of zinc oxide particles were used as precursors to produce zinc oxide networks by sintering, each with two different densities resulting in two different weight ratios of the PICNs. For each of these different networks, two subgroups were built: one involving the application of a phosphate-monomer-containing primer prior to the infiltration of Bis-GMA/TEGDMA and one without. Elastic modulus and flexural strength were determined by using the three-point bending test. Vertical substance loss determined by the chewing simulation was evaluated with a laser scanning microscope. There was a statistically significant influence of the type of precursor particles on the flexural strength and in some cases on the elastic modulus. The application of a primer lead to a significant increase in the flexural strength and in most cases also in the elastic modulus. A higher weight ratio of zinc oxide led to a significantly higher elastic modulus. Few statistically significant differences were found for the vertical substance loss. By varying the shape of the particles and the weight fraction of zinc oxide, the mechanical properties of the investigated PICN can be controlled. The use of a phosphate-monomer-containing primer strengthens the bond between the infiltrated polymer and the zinc oxide, thus increasing the strength of the composite.

## 1. Introduction

Dental restorative materials are under constant development to optimize their properties to be as close as possible to those of a natural tooth. For all restorative materials, the structural and mechanical properties of human enamel are considered ideal [1,2]. The material should be biocompatible in the oral environment and should not exhibit toxicity or cause allergic reactions [3]. To meet the multiple demands, ceramics and composites are often used. Ceramics have better mechanical and aesthetic properties, while dental composites are easier to process and repair intraorally [4].

Three major periods can be roughly distinguished in the development of dental composites to date: (1) Mid-1960s to late 1970s, with significant changes in curing properties (from self-curing and UV-curing to light-curing composites); (2) late 1970s to mid-2000s, with different filler modifications (from macro- and micro-filled to hybrid and nano-filled composites); and (3) mid-2000s to mid-2010s, with important resin modifications (from methacrylates to high-molecular-weight modified methacrylates, siloranes, and self-adhesive composites) [5]. There are hybrid, nano-hybrid, micro-filled, packable, ormocer-based, and flowable composites; compomers; and flowable compomers [6].

Conventional dental composites consist of an organic matrix into which inorganic filler particles are incorporated [7]. The organic matrix is composed of monomers, initiators, and inhibitors. Dimethacrylates such as bisphenol A glycidyl methacrylate (Bis-GMA) or urethane dimethacrylate (UDMA) are used as monomers. Comonomers such as dieethylene glycol dimethacrylate (DEGDMA) or trietylene glycol dimethacrylate (TEGDMA) are also added as crosslinkers and viscosity reducers. The inorganic filler particles consist of small glass, ceramic, and quartz particles mixed with fine silica particles. Attempts have been made to eliminate the weaknesses of conventional dental composites such as bacterial adhesion [7] and insufficient wear resistance [8] by adapting the individual components of the material, with nanofillers in particular modifying the material properties. For example, the reinforcement of dental composites with silica nanoparticles improves the mechanical properties compared to other composites [9].

Chairside CAD/CAM materials in dentistry can be divided into metal alloys, ceramics, resin, and resin-matrix ceramics [10]. Hybrid ceramics (a mixture of resin and ceramic) are a new category of chairside CAD/CAM materials, like the “polymer-infiltrated ceramic-network (PICN)” and high-performance polymers (HPPs) [10]. Polymer-infiltrated ceramic networks (PICNs) offer a promising approach to enhancing the material properties of composites. They are highly regarded in restorative dentistry, where the goal is to combine the desirable characteristics of ceramics and polymers [11]. This is crucial for optimizing material properties such as abrasion resistance and flexural strength, which are essential for withstanding chewing stress in dental applications. The composition of a PICN can be varied, and modifications in terms of type and weight ratios have been explored and tested for both the ceramic matrix and polymer components [12].

A well-known PICN used in dentistry consists of a feldspar matrix infiltrated with polymer. The ceramic feldspar matrix is porous and compressed; consists of silica, alumina, sodium oxide, and potassium oxide; and is infiltrated with a polymer blend consisting of UDMA/TEGDMA or BisGMA/TEGDMA [10]. In this PICN, the inorganic ceramic portion is 86 wt%, and the inorganic polymer portion, consisting of UDMA and TEGDMA, is 14 wt% [11]. It has been shown that this material has a clinically acceptable flexural and fracture strength [11]. In other studies on the fabrication of experimental PICNs, methacrylate-based resin was infiltrated into the porous silica ceramic network, or the PICN was prepared from a slurry of glass–ceramic powder, which was subsequently centrifuged, sintered, and infiltrated with urethane dimethacrylate [13,14].

With a mean elastic modulus of 40 GPa [15], it is slightly below that of tooth enamel with mean values of 50–85 GPa [16]. New approaches toward a PICN with zinc oxide as the network to be infiltrated have also been made. Zinc oxide is a white, odorless powder consisting of a chemical compound of a zinc atom and an oxygen atom.

To strengthen the adhesion of the polymer mixture to a ceramic network, primers can be used as adhesion promoters. In dentistry, for example, this is used for bonding to zirconia ceramic [17]. The use of primers containing a phosphoric acid monomer or a phosphate ester monomer improves the adhesion of resin to ceramic by creating chemical bonds between the components in addition to mechanical adhesion [18].

One aim of the present study was to investigate the influence of the weight ratio and the shape of the precursor particles on PICN material properties such as elastic modulus, flexural strength, and wear height loss.

The second aim of this study was to investigate influence of the application of a phosphate-monomer-containing primer on the mechanical properties of (PICNs) fabricated from zinc oxide.

The null hypothesis states that none of the three factors—the weight ratio, the shape of the precursor particles, and the phosphate-monomer-containing primer—exerts a statistically significant influence on the elastic modulus, flexural strength, and wear height loss.

## 2. Material and Methods

**Figure 1:**
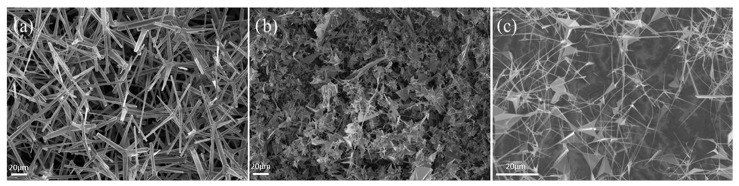
SEM images of different precursor particles: (**a**) tZnO powder, (**b**) sail-structured powder (**c**) sail-structured powder.

Tetrapodal zinc oxide and secondly zinc oxide particles with longer and thinner arms and a kind of sail structure were used to investigate the influence of the network. Both particle structures are shown in Figure 1.

The hexagonal rootite structure has four tetrapodal concave extensions arranged at an energetically favorable angle of 109.5° to each other [19,20].

### 2.1. Specimen and Precursor Preparation

**Figure 2:**
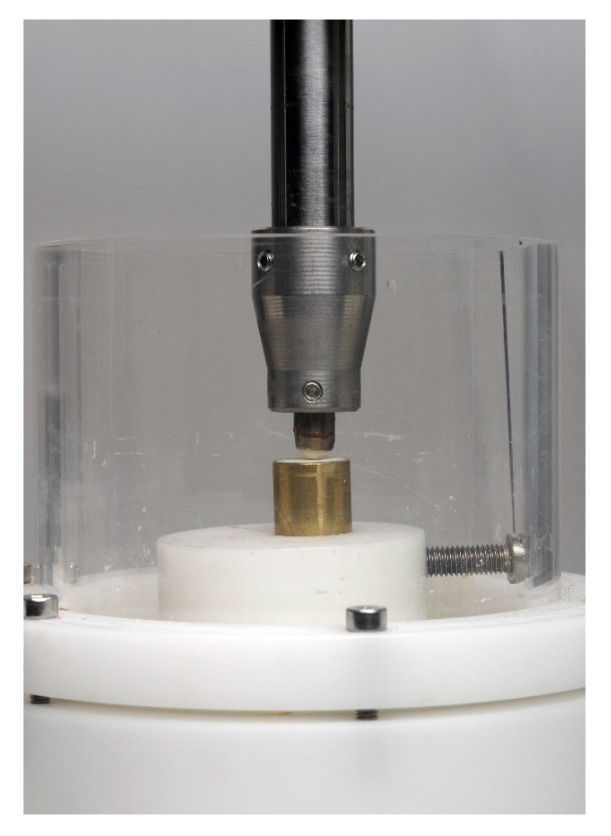
Sample positioned in a chamber of the chewing simulator. The sample is embedded in the metal sleeve, and the steatite sphere is positioned on the sample as an antagonist.

Four different ZnO networks were produced in the present study. In order to investigate the influence of the structure of the zinc oxide network on the mechanical properties of the PICNs, two groups of networks based on different kinds of zinc oxide particles—tetrapodal ZnO and sail-structured ZnO (Figure 2)—were produced. The different structures of tetrapodal zinc oxide can be produced by upscaling the zinc fraction during the fabrication [21]. It changes the size and thickness of the tetrapods. Additionally, networks with two different densities for each kind of zinc oxide network were produced in order to investigate the influence of the ceramic weight ratio on the mechanical properties of the PICNs. Test groups with their group codes are shown in Table 1.

For the specimens, the ZnO was weighed with a fine balance with a measurement uncertainty (of ±0.001 g) to obtain the final density for the zinc oxide network. A total polymer-to-ZnO ratio was prepared a low weight ratio at 40 wt%/60 wt% and high weight ratio at 25 wt.%/75 wt.%. Prior to the sintering process, the powder was shaped with a press and a pressure of 10 kN (PW; Paul-Otto Weber Laborpresstechnik, Remshalden, Germany) to discs of a thickness of 1.5 mm and a diameter of 15 mm and plates of a 2.1 mm thickness, length of 54 mm, and width of 10 mm. The pressed specimens were carefully placed on a refractory ceramic plate and sintering furnace at 1150 °C for 5 h (L3/12/B180; Nabertherm GmbH, Lilienthal/Bremen, Germany). Half of the specimens for each of the four different types of ZnO networks were treated with a phosphate-monomer-containing primer after sintering. For this purpose, the free volume of the ZnO networks was calculated, the appropriate amount of primer was dripped on with a pipette, and the further specimen preparation was interrupted until the visible liquid was evaporated. Subsequently, a monomer consisting of Bis-GMA and TEGDMA (ratio 60:40) (bisphenol A-glycidyl methacrylate; triethylene glycol dimethacrylate) was mixed with the photoinitiator and accelerator camperherquinone (0.25 wt.%) and ethyl4(dimethylamino)benzoate (0.25 wt.%) for 30 min in a dark fume hood with a magnetic stirrer. Subsequently, all specimens, also darkened, were stored with the stirred mixture for 30 min at 10 hPa in a vacuum unit and thus infiltrated. After infiltration, all specimens were cured for 40 s from each side using a light-curing lamp (Radii-cal LED curing light; SDI, Bayswater, VIC, Australia) providing a wavelength range of 440–480 nm at a distance of 0,8 cm. Finally, the discs were polished to 600 grit from both sides using a fine-polishing machine (EcoMet 250 Pro; Buehler, Lake Bluff, IL, USA) up to a thickness of 1.2 mm. The grinding process was carried out with water cooling at 180 revolutions per minute. The specimens were then blotted dry with paper towels. The plates were milled into the bars with dimensions of 2 × 2 × 25 mm and then also polished like the disks.

For each group, a total of 22 test specimens were prepared with two types of standardized specimens, i.e., 12 disks of each material for the chewing simulation and cross-sectional images, and 10 bars of each material for the three-point bending test.

Cross-sectional images of 4 disks of each material were made to evaluate the homogeneous infiltration of the polymer between the zinc oxide tetrapods. The cross-sections were examined using a scanning electron microscope (Zeiss Supra 55 VP, Jena, Germany). For this purpose, the cross-sections were first coated with a 10 nm thick gold layer (Leica EM QSG 100, Wetzlar, Germany), and then the images were acquired using an acceleration of 10 kV.

### 2.2. Material Science Experiments

#### 2.2.1. Three-Point Bending Test

The three-point bending test was performed on 10 specimens per group. The specimens were positioned on two supports (radius 5 mm) at a fixed distance (20 mm). A compression pin with a radius of 5 mm was moved onto the specimen at a crosshead speed of 2 mm per minute to a maximum depth of 6 mm, if no fracture occurred beforehand. Then, the force–deflection curve was recorded using the formula $\sigma =\frac{3Fl}{2bh^{2}}$ and the modulus of elasticity of the specimens was calculated using the formula $E=\frac{1}{4}\frac{l^{3}F}{bh^{3}s}$ . 

*F*: Force;

*s*: Deflection;

*l*: Distance between the two support points;

*b*: Width of the bar;

*h*: Height of the bar.

The clinical wear of materials is highly dependent on factors other than the material, e.g., the contact area [22]. For that reason, no power analysis was performed, as it is difficult to determine an adequate effect size that correctly takes the clinical relevance into account. Therefore, the number of specimens was chosen based on previous studies that investigated wear behavior with a number of specimens ranging from 5 to 10 per group [23]. Data were statistically analyzed using statistical software and a significance level of α = 0.05 (SPSS version 20, IBM, Armonk, NY, USA). The Shapiro–Wilk test did not contradict a normal distribution for the data from the three-point bending test. A three-way analysis of variance (ANOVA) was performed with the factors weight ratio, precursor particles, and primer, followed by two-way analysis of variance and one-way analysis of variance if necessary.

#### 2.2.2. Wear Evaluation

The 64 specimens for the chewing simulation were embedded in a polyester resin in a brass tube and placed in a chewing simulator. After embedding the polished specimens, eight specimens of each material were placed in the chewing simulator (CS-4 SD-Mechatronik, Feldkirchen-Westerham, Germany) and loaded with 5 kg for 1.2 million cycles, using a steatite sphere with a diameter of 6 mm as an antagonist. Wear height loss was examined for each specimen after the chewing simulation with a laser scanning microscope (vk-x 100, Keyence, Osaka, Japan) using a laser with a wavelength of 658 nm and evaluated with a software program (VK-H1XAD, Keyence).

Data were also statistically analyzed using statistical software and a significance level of α = 0.05 (SPSS version 20, IBM, Armonk, NY, USA). For the wear height loss data, the Shapiro–Wilk test did not contradict a normal distribution. A three-way analysis of variance (ANOVA) was performed with the factors weight ratio, precursor particles, and primer, followed by two-way analysis of variance and one-way analysis of variance if necessary.

The specimen was positioned in a chamber of the chewing simulator. The specimen was embedded in the metal sleeve, and the steatite sphere was positioned on the specimen as an antagonist.

## 3. Results

### 3.1. Three-Point Bending Test

**Figure 3:**
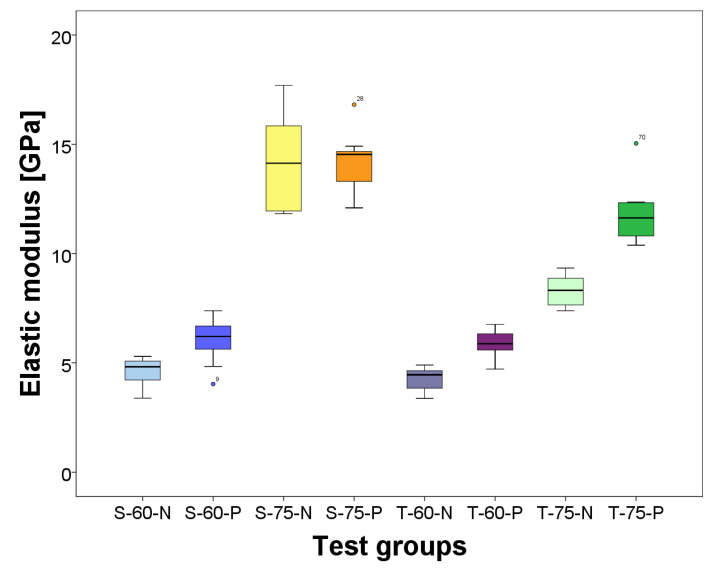

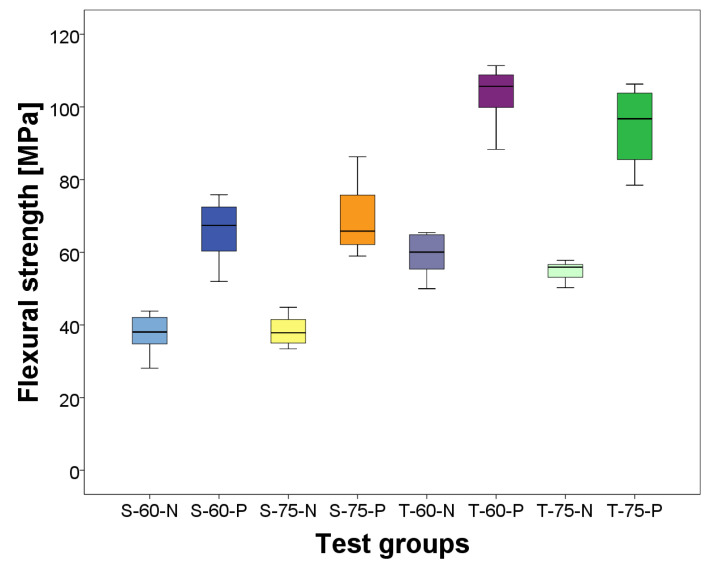
Boxplots of elastic modulus and flexural strength of all test groups.

The means, standard deviations, and statistically significant differences of the elastic modulus measured by the three-point bending test are given in Table 2 and those of the flexural strength in Table 3. Boxplots of both properties are shown in Figure 3.

The weight ratio makes a significant difference in the elastic modulus for both precursor particles and with and without primer. The groups with the higher weight ratio always obtain higher values with respect to the groups with low weight ratio. In the case of flexural strength, the weight ratio only has a significant effect only on the PICNs from tZnO: T-60-N and T-60-P are significantly higher than T-75-N and T-75-P, respectively. For the PICNs with a sail structure, there are no significant differences in terms of the weight ratio. The precursor particles, regardless of whether a primer was used or not, have no significant effect on the elastic modulus at a low weight ratio but do at a high weight ratio. Here, the high-weight-ratio groups and the PICNs with a sail structure, i.e., groups S-75-N and S-75-P, T-75-N, have higher values than the PICNs with tZnO: T-75-N and T-75-P. The precursor particles have a significant influence on the flexural strength. All PICNs from tZnO achieved higher values with respect to the groups with a sail structure regardless of the primer and weight ratio. The primer had a significant effect on the elastic modulus for all but one group: the specimens with the sail-structure high-density PICNs (S-75-N and S-75-P). For the other groups, the specimens with primer, i.e., groups S-60-Pand T-60-P, obtained the higher values. Bonding of the primer to the zinc oxide increased the flexural strength statistically significantly, both in terms of weight ratio and precursor particles. All specimen groups with primer, i.e., groups S-60-P, S-75-P, T-60-P, and T-75-N, achieved higher values.

### 3.2. Wear Evaluation

The wear height loss of the specimens after the chewing simulation is shown in Table 4.

**Figure 4:**
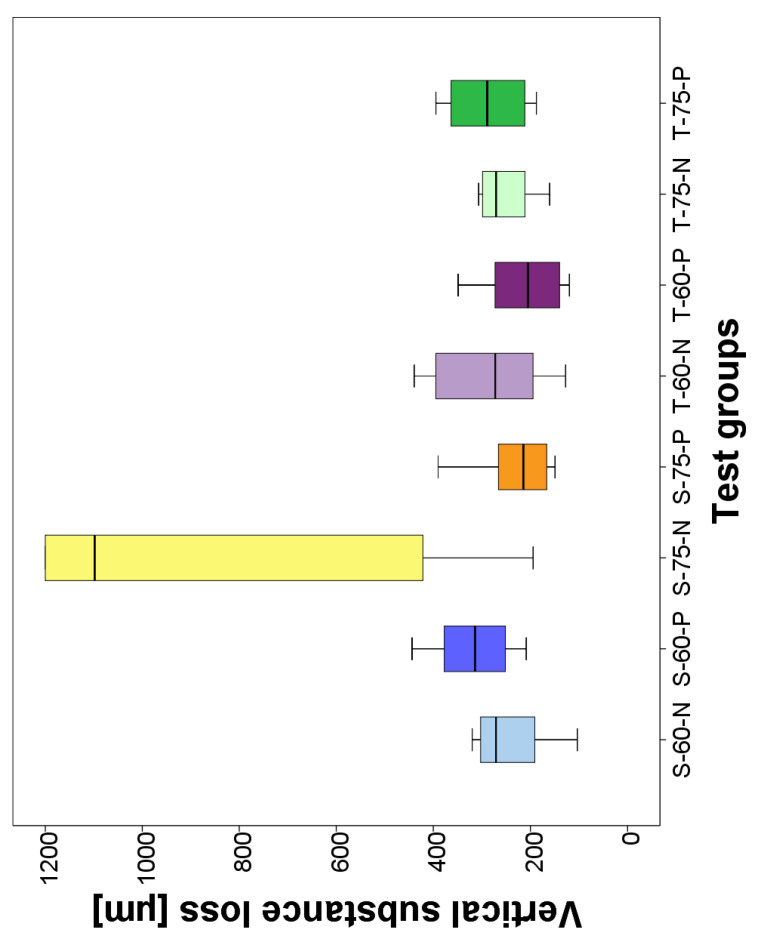
Boxplot of vertical substance loss after chewing simulation.

The mean values of the wear height loss ranged from 213 µm to 317 µm with the exception of the PICNs with a sail structure at a high weight ratio without primer, which exhibited the highest wear height loss with 854 µm ± 437 µm. In this group, some specimens were worn through and therefore were included in the statistical evaluation with a heigh loss of 1200 µm. No system was recognizable in the statistical differences in wear height loss. The Boxplot of vertical substance loss is shown in Figure 4.

## 4. Discussion

Since all three factors, weight ratio, the choice of precursor particles, and the use of a phosphate-monomer-containing primer, had a statistically significant effect on the investigated properties, the null hypothesis was rejected.

The choice of weight ratio, precursor particles, and primer showed a significant difference in the properties. The elastic modulus was significantly higher in the specimens with the higher weight ratio than in those with a weight ratio of 1.3 g/cm^3^. The higher the weight ratio in the material, the more resistant the network seems to become to mechanical loading [24]. This could be because higher weight ratios lead to more tetrapod ends, and such materials can resist more stress and strain than those with fewer tetrapod ends. As the proportion of zinc oxide content is higher, the specimens become stiffer. When the concentration of the filler exceeds the optimum value, the total surface area of the tetrapods increases, but the weight percentage of the matrix content decreases. As a result, the bond strength is weakened, and the load is not uniformly transferred to the entire specimen, leading to a decrease in elastic modulus [25]. The specimens made of sail-structured particles exhibited higher values within the two weight ratios than those made of tZnO. It was also shown that in composites with filler particles, the smaller filler particles increase the elastic modulus [26]. This can also be seen in the results since the sail structures are smaller in diameter than the tZnO and achieve the higher values.

Sail structures, due to their sail formation, have additional, larger areas in the sail formation region that can accommodate strains better than when these sails are not present. The sail formation of the tetrapods in the sail structure probably allows better absorption of stress and strain forces in the sail region but is more fragile due to the much thinner tetrapod arms at the transitions from sail to sail compared to the tZnO because the network is weaker as a result. The arms of the sail structure are significantly thinner than those of the tZnO, which causes them to break first and reduces the bending strength [27]. The values of flexural strength support this theory, since here, opposite to the values for elastic modulus, the PICNs made of tZnO in this study achieve higher values than the PICNs with a sail structure. Nevertheless, the material appears to be too flexible overall, as the maximum mean elastic modulus values here of 14.2 GPa (PICNs with a sail structure, 2.3 g/cm^3^, with primer) are considerably lower than those of a comparable PICN, described in the introduction, which achieves average values of 30 GPa [11]. In addition to the mechanical adhesion between the polymer matrix and the zinc oxide particles, the primer also ensures chemical bonding. The primer provides a general strengthening of the framework so that the specimens with primer reach higher values than those without primer, depending on the weight ratio and precursor particles.

For flexural strength, it was clearly evident that the materials additionally infiltrated with a primer showed significantly higher values than the specimens without primer. Similar to the elastic modulus, the primer significantly strengthens the overall framework by improving the bonding of the tetrapods to the polymer, making it more difficult for them to pull out of the network. After subdividing the materials into those with and without primer, the PICNs made of tZnO exhibit higher values for flexural strength than the PICNs with a sail structure. This can be explained by the fact that the diameters of the individual tetrapods are thicker than those of the sail structure and thus more stable. Overall, a ceramic network provides reinforcement of the framework, as was also shown in another study [11]. The maximum flexural strength value of 102.8 MPa achieved in this study is significantly lower than those of the comparable PICNs mentioned at the beginning with values of 131.1–159.9 MPa [11,28]. The ISO standard for materials in this category is 50 MPa [29]. This value was not achieved by all PINCs produced in this study.

The choice of precursor particles for the network has a significant impact on the material. There are currently no studies on how other tetrapodal precursor particles behave in such experiments. It would be interesting to test how the material properties change when the weight percentages of the precursor particles are further altered and how other tetrapodal zinc oxides with different thicknesses and shapes of the tetrapod arms would behave. In addition, how the sintering temperature of the precursor particles affects the network could be an interesting topic for further studies. The temperature must be high enough for the zinc oxide to sublimate and the arms of the tetrapods to fuse to form a stable network. Another important effect is that oxygen defects form on the zinc oxide surface, making the networks hydrophilic and easy to infiltrate. The temperature of 1150 °C and time of 5 h chosen in this study have so far yielded reliable results for a stable network in some trials [21]. Therefore, these parameters were not changed in the present study. Changing the sintering parameters could also have a significant influence on the stability of the network, for which there are currently no studies for the precursor particles used in this study. For other precursor particles, consisting of sodium aluminum silicate, it could be demonstrated that the sintering temperature has a significant influence on the stability of the network [30].

The wear height loss after the chewing simulation somewhat reflects the material behavior at depth. The wear height loss values range from 213 µm to −317 µm (Table 4), with the exception of the PICNs with a sail structure without primer with the high weight ratio. Although the weight ratio, the precursor particles, and the primer each have a significant influence on the wear height loss, no order system can be detected in the abrasiveness of the materials. Presumably, for all materials, the bonds are broken in a similar way and simply polished over by antagonists in the course of the chewing simulation. By far the largest wear height loss was observed for the sail-structured particles in the high weight ratio without primer with a value of 854 µm ± 437 µm (Table 4). This could be due to the fact that the material was not fully light-cured to the center during production due to the sail formation of the material and the additional high density. The sails may have shielded the polymer from the light, making the material slightly softer on the inside and thus much less wear-abrasive. To avoid the incomplete polymerization of the light-curing composite, it might be beneficial to use a dual or chemically curing composite. In one study, self-curing dual composites were shown to cure uniformly to a depth of 6 mm [31]. This could potentially allow the material to cure under the sails as well, which was previously thought to be prevented by shielding. Under similar conditions of the chewing simulation, one study found a mean wear height loss of 157 µm for a PICN (Vita Enamic), 202 µm for lithium disilicate, and 191 µm for zirconia reinforced lithium disilicate. The values, in particular of the PICNs, are significantly lower than those of the wear height loss of the PICNs determined in the current study. This could indicate that these PICNs are still too small overall and break too easily [32]. Overall, it should be noted that flat specimens have a higher material loss than anatomical specimens, as the stress distribution is greater [33]. In this study, however, plates were used for the chewing simulation, as they were all individually pressed, infiltrated, and polished by hand during production. This would have been difficult to achieve with a crown shape. In comparable studies, plates were also used instead of crowns for the chewing simulation [11,34].

This study has some limitations which need to be considered. One of them is the above-mentioned use of plates instead of crowns as specimens in the chewing simulation. In addition, the tests carried out do not reflect the stress conditions in the mouth. Another limitation is that the specimens were prepared by hand and are therefore susceptible to minimal deviations from one another. In addition, it was not possible to compare the PICNs produced here directly with the described PICNs in the experiments but only with test values from other studies.

## 5. Conclusions

Taking the conditions and results of this study into account, the following conclusions, which only apply to the materials used in this study, can be drawn:

1. The use of a phosphate-monomer-containing primer improves the elastic modulus and flexural strength of the tested PICNs.
2. A higher weight ratio significantly increases the flexural strength, while the formation of sail structures in the tested PICNs improves the elastic modulus.
3. To optimize these PICN material properties from a dental point of view, thicker tetrapods should be used than those in this study, which cause sail formation.

## Figures and Tables

**Table 1:** Group codes of test groups.

Precursor Particle	Zinc Oxide Weight Ratio	Primer Application	Group Code
Sail structure	60 wt.%	No	S-60-N
		Yes	S-60-P
	75 wt.%	No	S-75-N
		Yes	S-75-P
Tetrapodal structure	60 wt.%	No	T-60-N
		Yes	T-60-P
	75 wt.%	No	T-75-N
		Yes	T-75-P

**Table 2:** Mean elastic modulus (standard deviation) given in GPa of all investigated PICNs based on ZnO. Statistically different elastic moduli (*p* ≤ 0.05) are indicated by different uppercase letters (ceramic content), lowercase letters (primer), or lowercase Greek letters (precursor particles).

Precursor Particles	Functionalization with Primer		60 wt.%	75 wt.%
sail structure	No	Mean SD	4.6 ^B^_b_^α^ (0.6)	14.2 ^A^_a_^α^ (2.1)
sail structure	Yes	Mean SD	6.0 ^B^_a_^α^ (1.0)	14.2 ^A^_a_^α^ (1.3)
tZnO	No	Mean SD	4.3 ^B^_b_^α^ (0.5)	8.3 ^A^_b_^β^ (0.7)
tZnO	Yes	Mean SD	5.9 ^B^_a_^α^ (0.6)	11.8 ^A^_a_^β^ (1.4)

**Table 3:** Mean flexural strength (standard deviation) given in MPa of all investigated PICNs based on ZnO. Statistically different flexural strengths (*p* ≤ 0.05) are indicated by different uppercase letters (ceramic content), lowercase letters (primer), or lowercase Greek letters (precursor particles).

Precursor Particles	Functionalization with Primer		60 wt.%	75 wt.%
sail structure	No	Mean SD	37.8 ^A^_b_^β^ (5.0)	38.3 ^A^_b_^β^ (3.8)
sail structure	Yes	Mean SD	65.3 ^A^_a_^β^ (8.2)	69.4 ^A^_a_^β^ (9.8)
tZnO	No	Mean SD	59.4 ^A^_b_^α^ (5.2)	55.1 ^B^_b_^α^ (2.5)
tZnO	Yes	Mean SD	102.8 ^A^_a_^α^ (8.5)	94.2 ^B^_a_^α^ (10.3)

**Table 4:** Mean vertical substance loss (standard deviation) given in µm of all investigated PICNs based on ZnO. Statistically different vertical substance loss (*p* ≤ 0.05) is indicated by different uppercase letters (ceramic content), lowercase letters (primer), or lowercase Greek letters (precursor particles).

Precursor Particles	Functionalization with Primer		60 wt.%	75 wt.%
sail structure	No	Mean SD	244 ^B^_a_^α^ (76)	854 ^A^_a_^α^ (437)
sail structure	Yes	Mean SD	317 ^A^_a_^α^ (82)	229 ^B^_b_^α^ (80)
tZnO	No	Mean SD	286 ^A^_a_^α^ (116)	254 ^A^_a_^β^ (56)
tZnO	Yes	Mean SD	213 ^A^_a_^β^ (81)	289 ^A^_a_^α^ (84)

## Data Availability

The original contributions presented in the study are included in the article, further inquiries can be directed to the corresponding author.
